# Supplementary material for: Simultaneous capturing phonon and electron dynamics in MXenes
Source: Nat Commun. 2022 Dec 22;13:7900. doi: 10.1038/s41467-022-35605-7 (PMC9780317; doi:10.1038/s41467-022-35605-7)
Supplement: Supplementary file 1 — Supplementary Information [file 41467_2022_35605_MOESM1_ESM.pdf]

Supplementary Information

Simultaneous capturing phonon and electron dynamics in MXenes

Qi Zhang[1], Jiebo Li[2*], Jiao Wen[3], Wei Li[4], Xin Chen[4], Yifan Zhang[2], Jingyong Sun[3], Xin Yan[5], Mingjun Hu[3], Guorong Wu[1], Kaijun Yuan[1,6*], Hongbo Guo[3*], Xueming Yang[1,6,7]

1 State Key Laboratory of Molecular Reaction Dynamics and Dalian Coherent Light Source, Dalian Institute of Chemical Physics, Chinese Academy of Sciences, 457 Zhongshan Road, Dalian 116023, P.R. China.

2 Institute of Medical Photonics, Beijing Advanced Innovation Center for Biomedical Engineering, School of Biological Science and Medical Engineering, Beihang University, Beijing 100191, P.R. China.

3 School of Materials Science and Engineering, Beihang University, Beijing, 100191, P.R. China.

4 GuSu Laboratory of Materials, Suzhou 215123, Jiangsu, China.

5 School of Mechanical Engineering and Automation, Beihang University, Beijing 100191, P. R. China.

6 Hefei National Laboratory, Hefei 230088, China.

7 Department of Chemistry, College of Science, Southern University of Science and Technology, Shenzhen 518055, P. R. China.

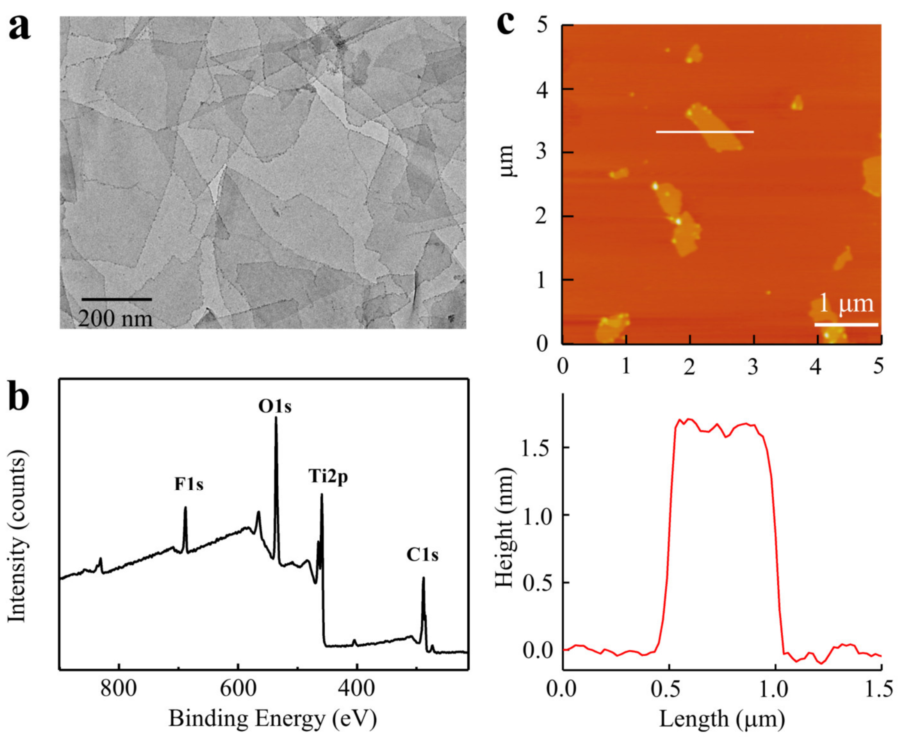

Supplementary Figure 1. Characterization of $Ti_3C_2T_x$. (**a**) TEM image. (**b**) XPS spectrum. (**c**) AFM image of $Ti_3C_2T_x$ on silicon (above) and a corresponding height of the flake (blow).

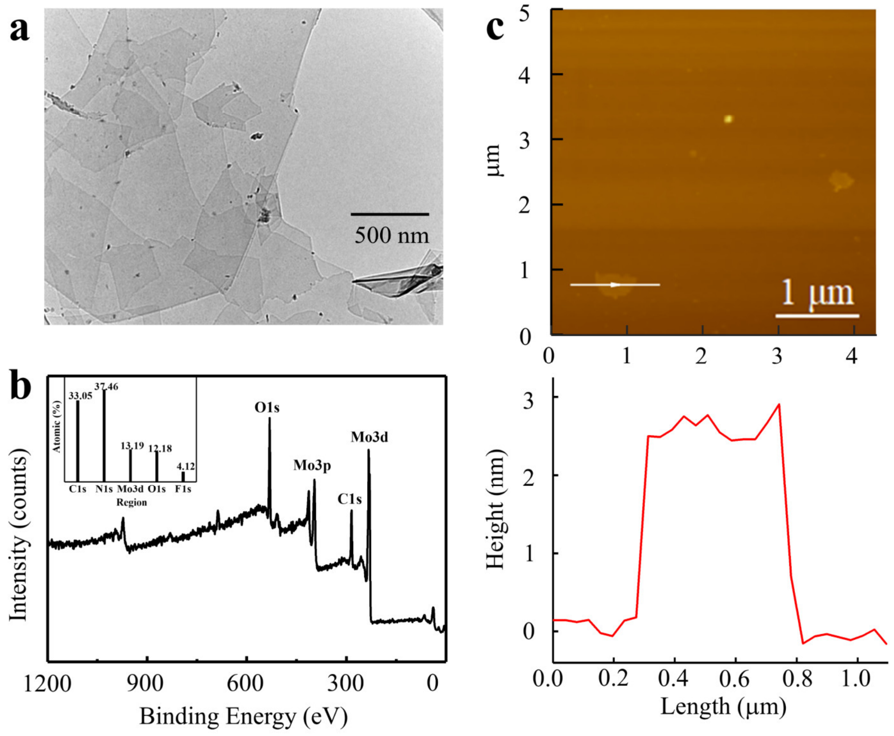

Supplementary Figure 2. Characterization of Mo_2CT_x. (**a**) TEM image. (**b**) XPS spectra. (**c**) AFM image of $Ti_3C_2T_x$ on silicon (above) and a corresponding height of the flake (blow).

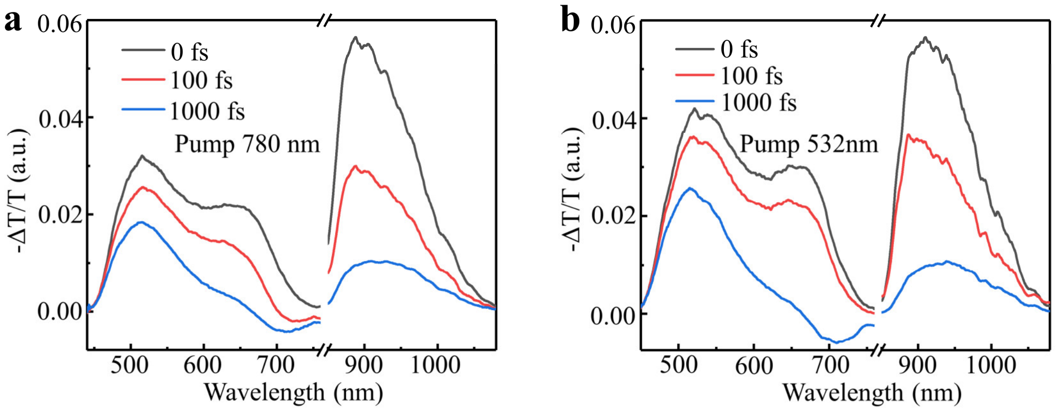

Supplementary Figure 3. Transient spectra of $Ti_3C_2T_x$ at various waiting times. (a) Pump 780 nm. (**b**) Pump 532 nm.

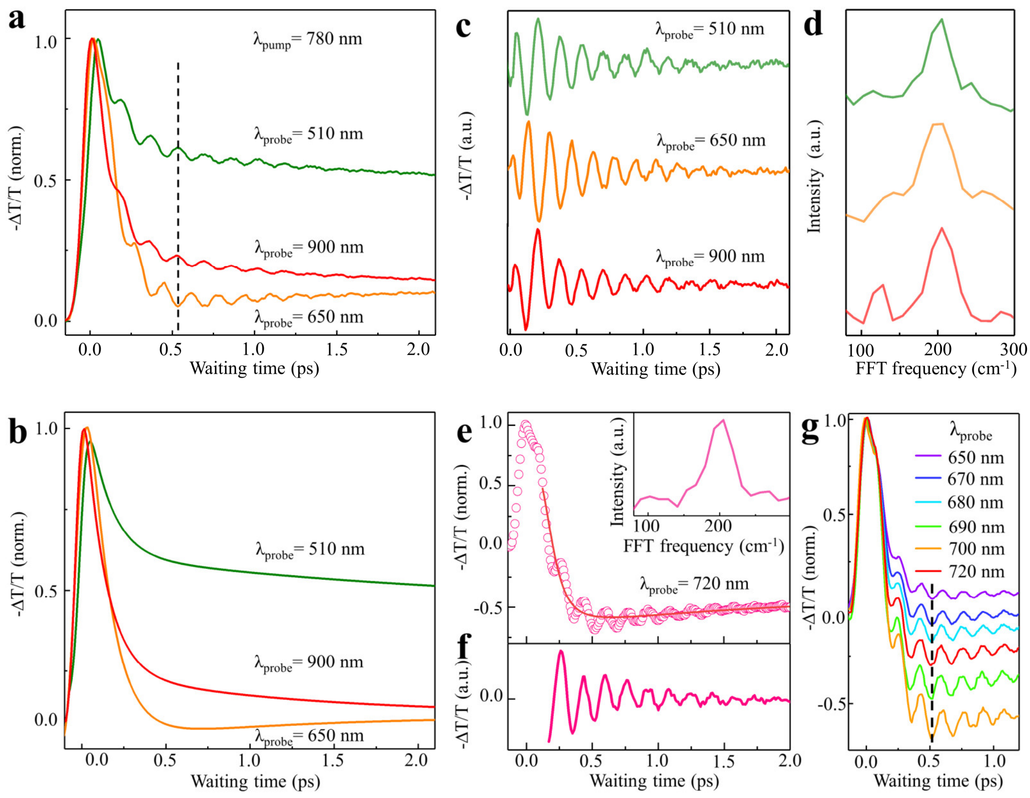

Supplementary Figure 4. Electronic and CP dynamics of $Ti_3C_2T_x$ at various probe regions with pumping 780 nm. (**a**) Trace of dynamics monitored at 510 nm, 650 nm, and 900 nm. (**b**) Fitting results. (**c**) CP dynamics were monitored at 510, 650, and 900 nm. (**d**) FFT vibration spectra during 0.1-1.0 ps with probe 510, 650, and 900 nm. (**e**) Trace dynamics monitored at 720 nm and the FFT spectrum in insert with probe 720 nm. (**f**) The CP dynamic was monitored at 720 nm. (**g**) Trace of dynamics monitored at plasmon band (probe 650, 670, 680, 690, 700, 720 nm).

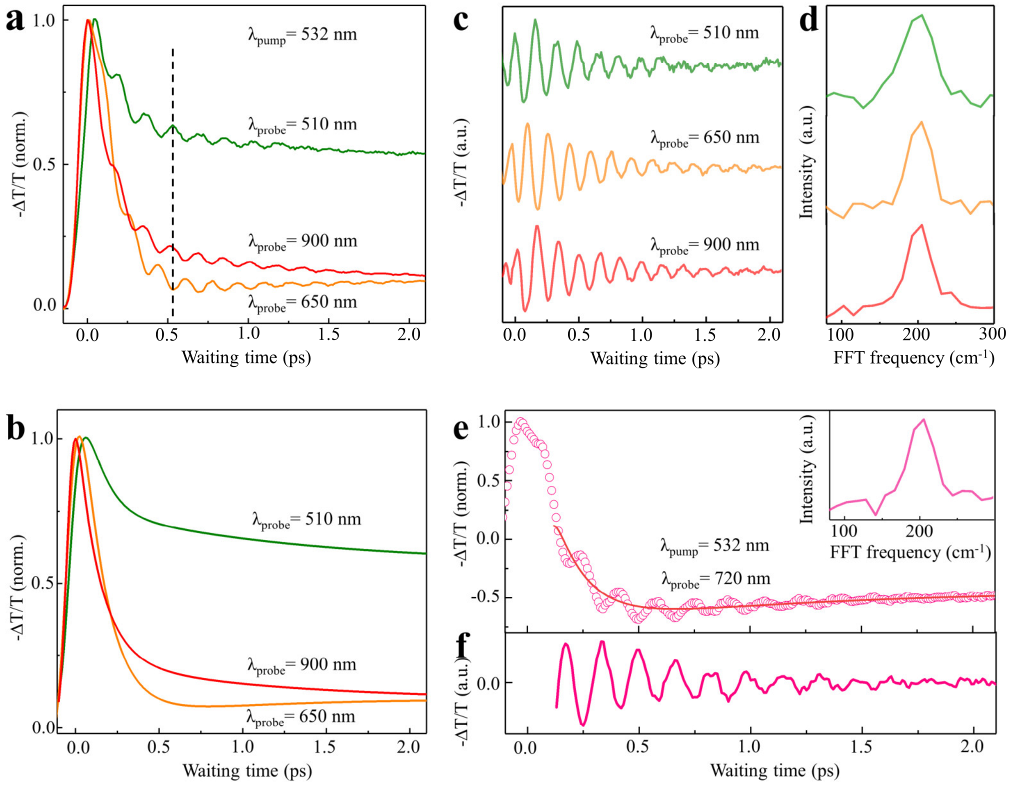

Supplementary Figure 5. Electronic and CP dynamics of $Ti_3C_2T_x$ at various probe regions with pumping 532 nm. (**a**) Trace of dynamics monitored at 510 nm, 650 nm, and 900 nm. (**b**) Fitting results. (**c**) CP dynamics were monitored at 510, 650, and 900 nm. (**d**) FFT vibration spectra during 0.1-1.0 ps with probe 510, 650, and 900 nm. (**e**) Trace dynamics monitored at 720 nm and the FFT spectrum in insert with probe 720 nm. (**f**) The CP dynamic was monitored at 720 nm.

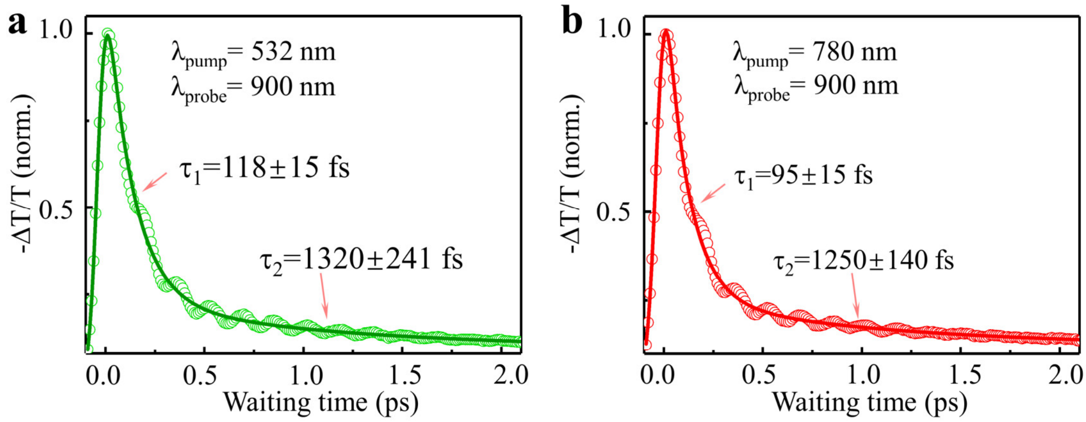

Supplementary Figure 6. The electronic dynamics of $Ti_3C_2T_x$ at probe 900 nm with fitting by two-exponential-decay. (**a**) Pump 532 nm. (**b**) Pump 780 nm.

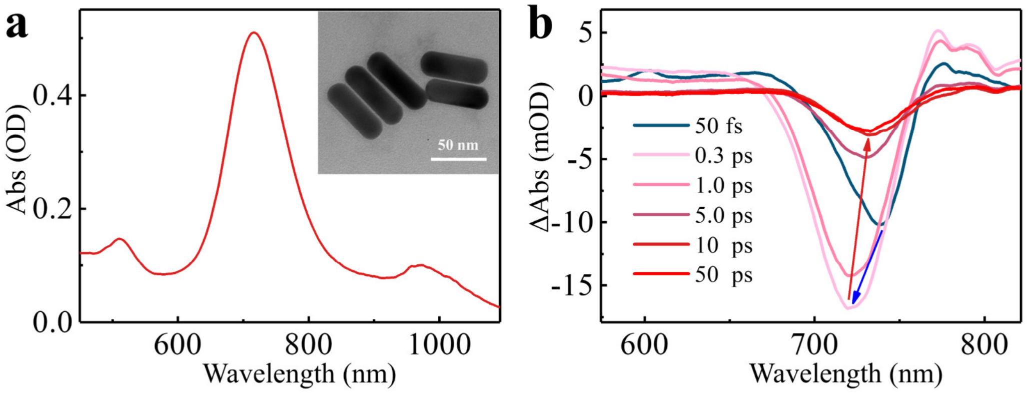

Supplementary Figure 7. Stead-state absorption spectrum and TA spectra of GNRs. (**a**) An UV-visible absorption spectrum of the GNRs in an aqueous solution exhibits a longitudinal SP absorption peak at ~720 nm. And an inset shows a TEM image of GNRs. (**b**) Transient spectra of GNR at indicated waiting times after 750 nm excitation.

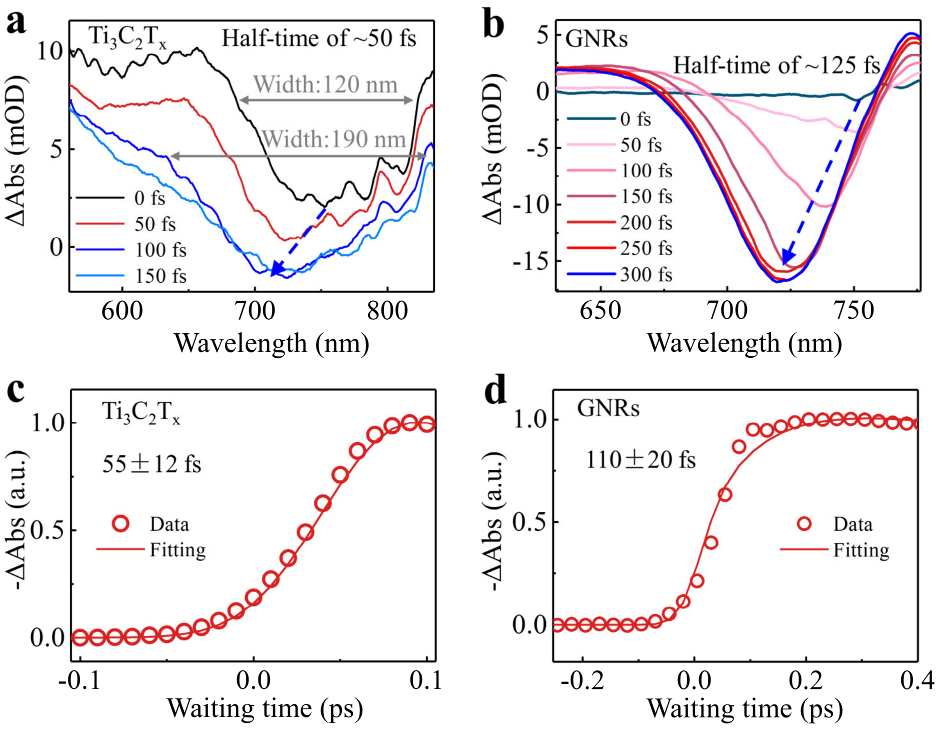

Supplementary Figure 8. The time constant of electron-electron scattering. (**a**) Transient spectra of $Ti_3C_2T_x$ film at indicated waiting times after 790 nm excitation. (**b**) Transient spectra of GNRs in aqueous solution at indicated waiting times after 750 nm excitation. (**c**) The dynamic evolution of $Ti_3C_2T_x$ film probed at 903 nm after 780 nm excitation. (**d**) The rise dynamic evolution of GNRs in aqueous solution probed at 720 nm after 750 nm excitation.

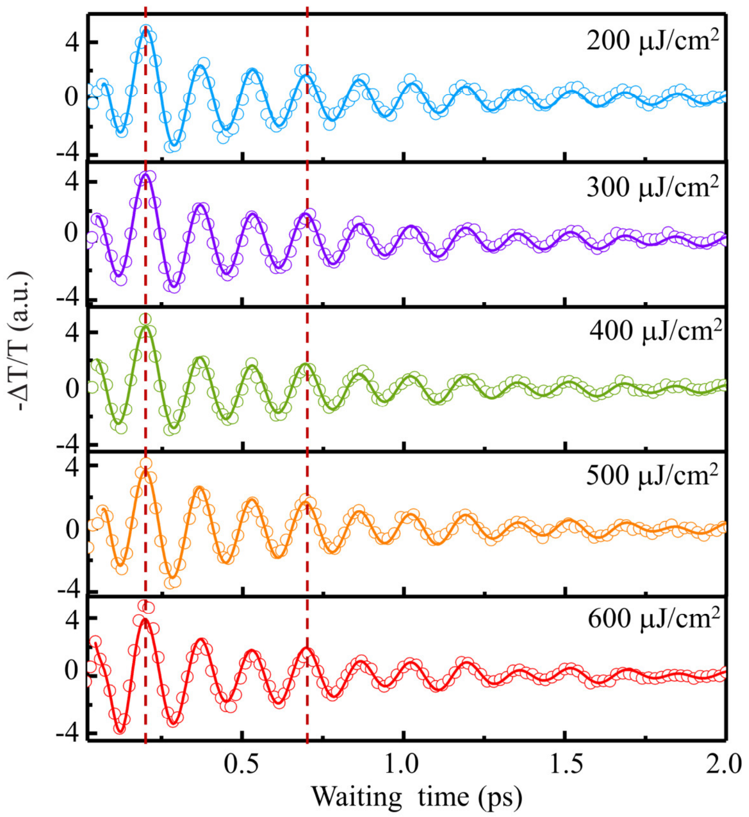

Supplementary Figure 9. Pump fluences-dependents CP dynamics extracted from Fig. 3c probed at 900 nm after 780 nm excitation.

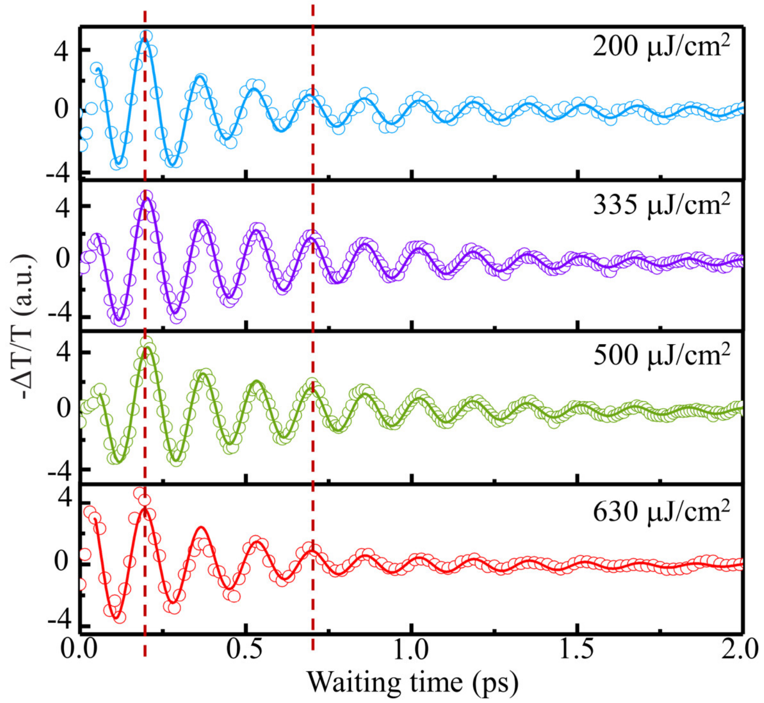

Supplementary Figure 10. Pump fluences-dependents CP dynamics extracted from Fig. 3d probed at 900 nm after 532 nm excitation.

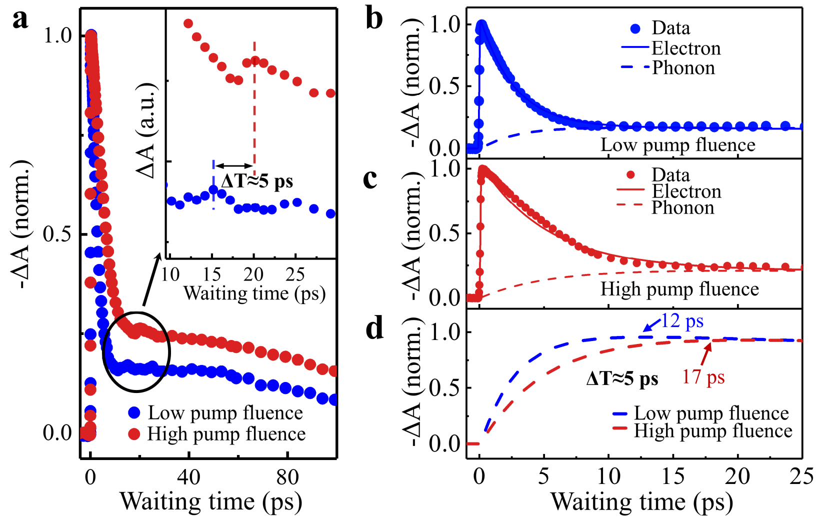

Supplementary Figure 11. The dynamic evolution of GNRs in aqueous solution probed at 720 nm after 750 nm excitation. (**a**) Pump-probe signals were monitored with low and high pump fluences. The insert is the enlarged view of dynamic evolution during10-30 ps. The dashed blue and red lines mark the peak positions of the oscillations to the two pump fluence, respectively. (**b**) A dynamics evolution with a low pump fluence of 30 nJ/pulse. (**c**) A dynamic evolution with a high pump fluence of 120 nJ/pulse. (**d**) Compared fitting phonon dynamics features with the low and the high pump fluence. The solid line and dashed line are fits to the electron and phonon dynamics, respectively. The circles are experimental data.

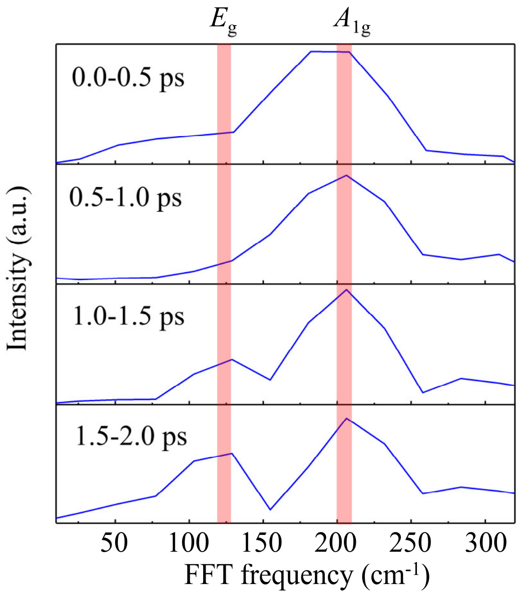

Supplementary Figure 12. Time-resolved FFT spectrum of $Ti_3C_2T_x$ with pump 780 nm probe 900 nm.

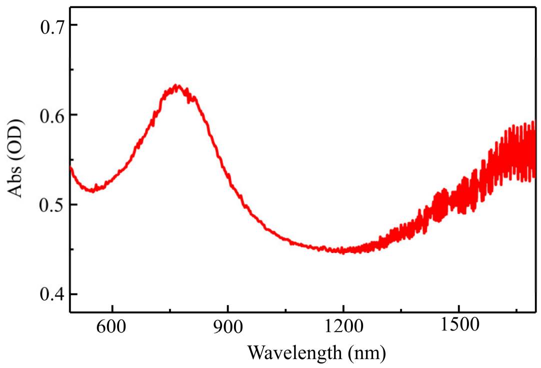

Supplementary Figure 13. Absorption spectrum of $Ti_3C_2T_x$ film with the near-infrared region.

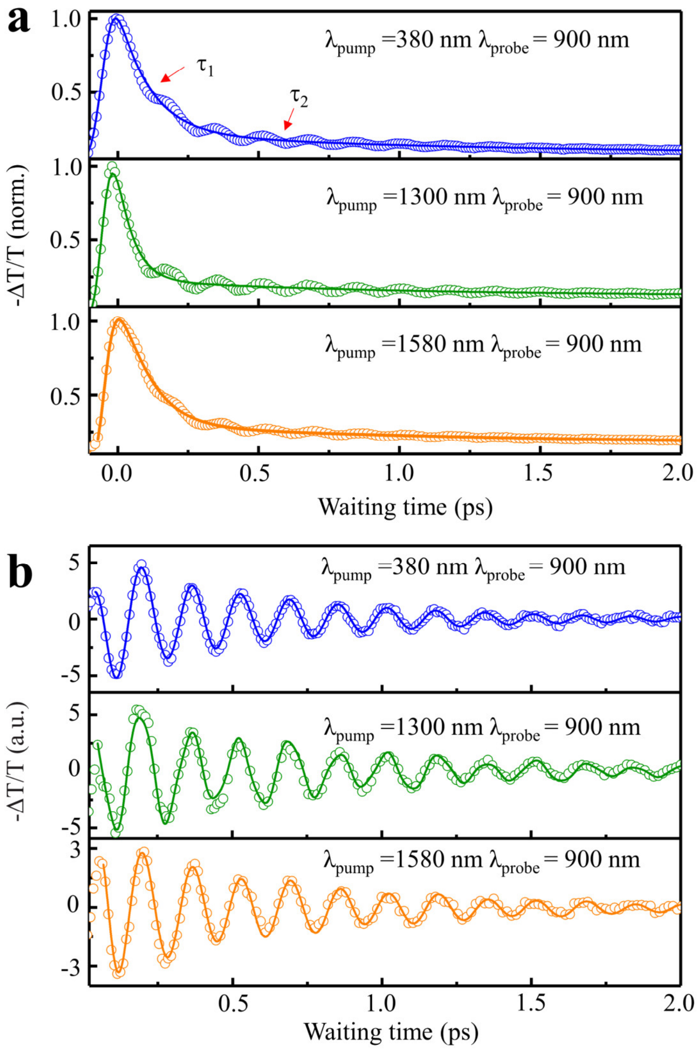

Supplementary Figure 14. Dynamics of $Ti_3C_2T_x$ were monitored at 900 nm with pumping 380 nm 1300 nm, and 1580 nm, respectively. (**a**) Electronic dynamics. (**b**) CP dynamics.

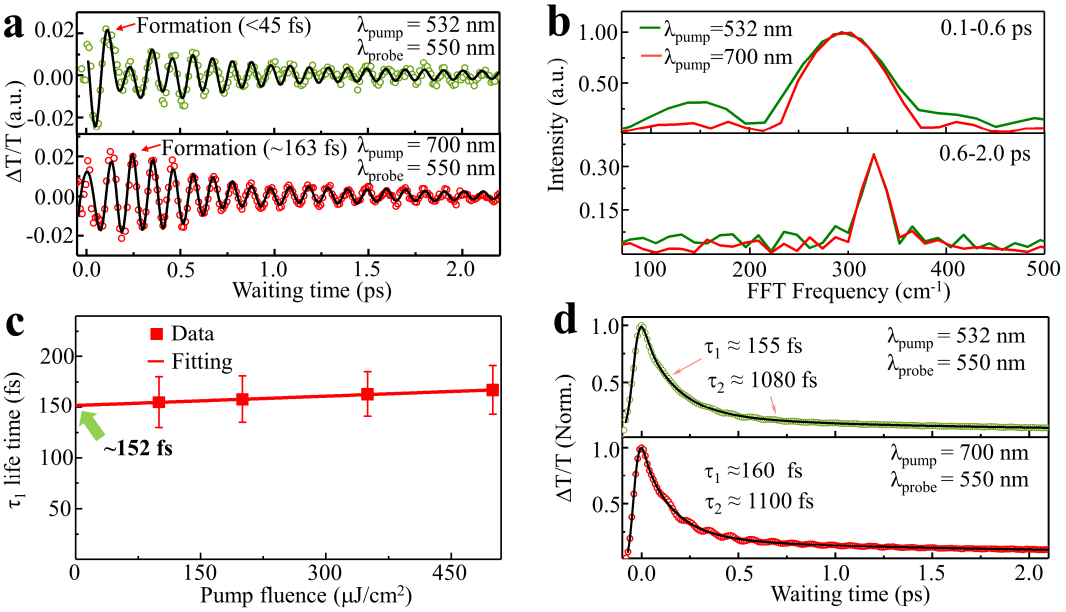

Supplementary Figure 15. Ultrafast electronic excitation dynamics, CP dynamics and time-resolved IVS for Mo_2CT_x. (**a**) CP dynamics (circles) and fitting results (black lines) for probing at 550 nm with different excitation wavelengths. (**b**) FFT vibration spectra obtained from vibration dynamics data during 0.1-0.6 ps and 0.6-2.0 ps with pumping at 532 nm and 700 nm at 250 μJ/cm^2. (**c**) Time constants of pump fluence dependence with pumping at 700 nm and probing at 550 nm from Supplementary Fig. 24. The error bars represent standard deviation. (**d**) Electronic dynamics (green and red circles) and fitting results (black lines) with a two-exponential decay.

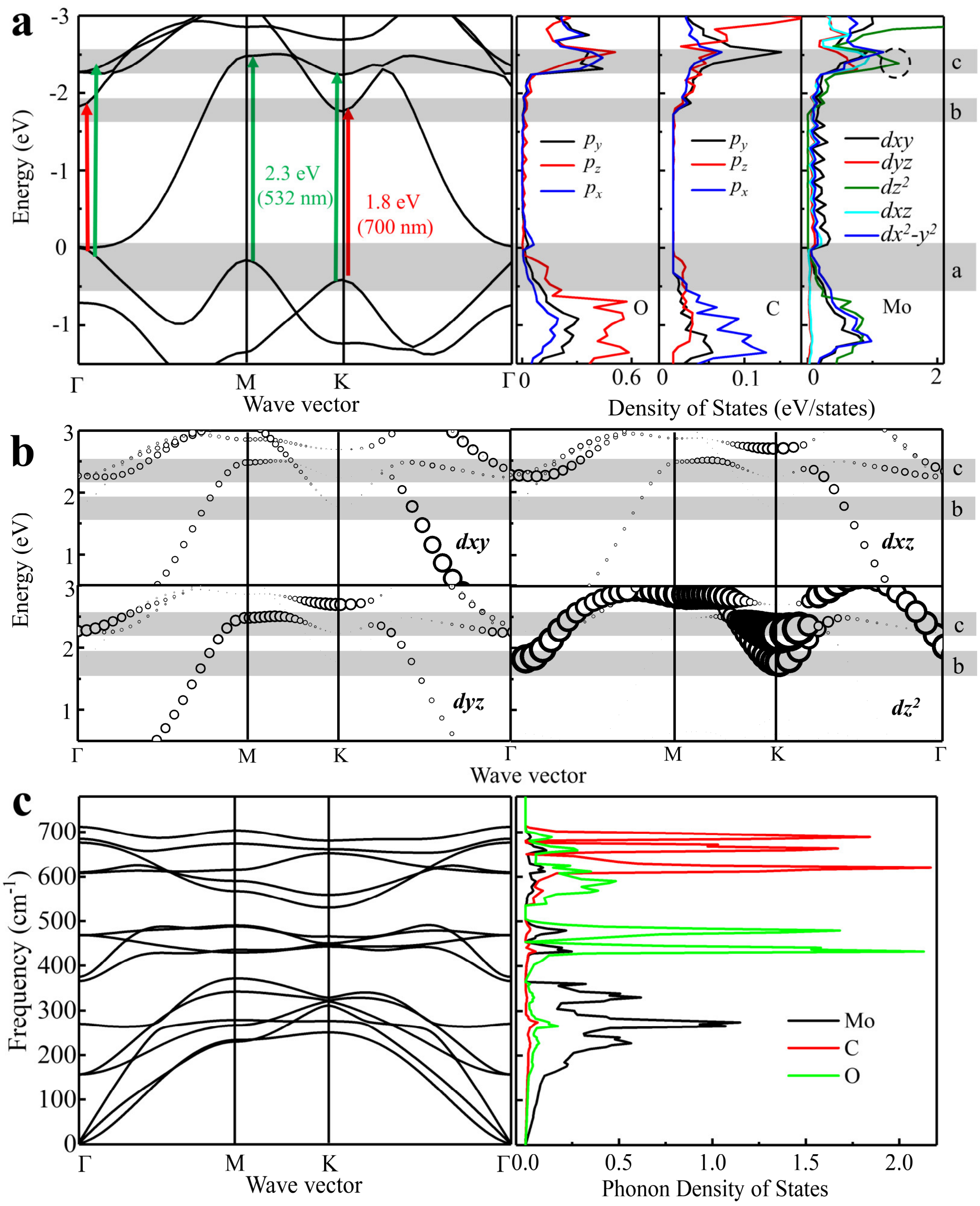

Supplementary Figure 16. The standard DFT calculated band structures and density of states (DOS) of Mo_2CT_x (Mo_2CO_2). (**a**) Electron structures and DOS, marked around -0.2 eV as the band, around 1.8 eV as the b band (main contribution of Mo dz^2 orbitals), around 2.3 eV as the c band (Contribution of Mo dz^2, *dxy*, *dxz*, *dyz*, dx^2-y^2). The different colored arrows indicate possible electronic transitions after excitation with different pump wavelengths. (**b**) Fatband of *dxy*, *dxz*, *dyz* and dz^2 orbitals (The diameters of the circles represent the intensity of DOS). (**c**) The phonon dispersions band and DOS.

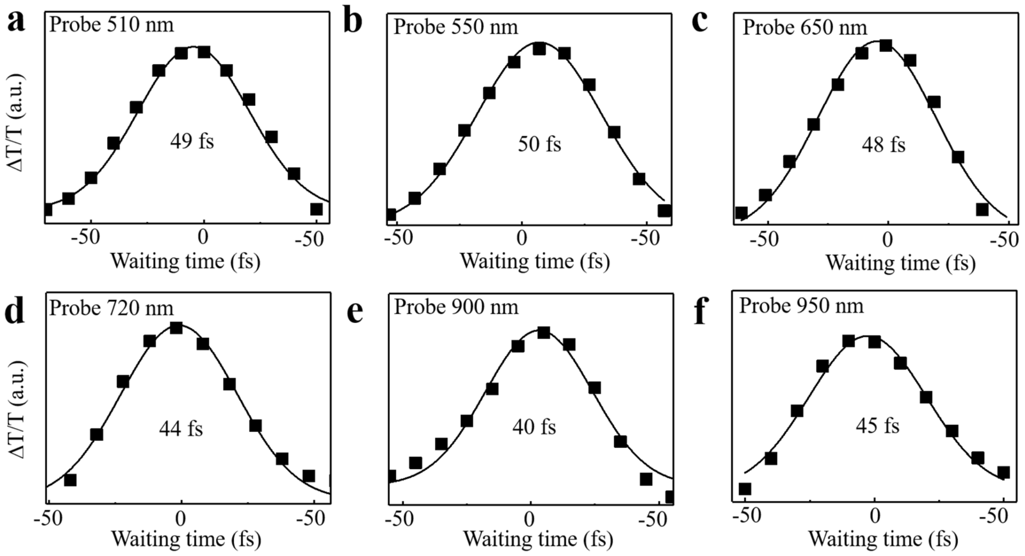

Supplementary Figure 17. Instrument response functions measurement with substrate (CaF_2) at various probe wavelengths. (**a**) 510 nm, 49 fs; (**b**) 550 nm, 50 fs; (**c**) 650 nm, 48 fs; (**d**) 720 nm, 44 fs; (**e**) 900 nm, 40 fs; (**f**) 950 nm, 45 fs. The circles are experimental data and lines are fitted results with Gaussian function.

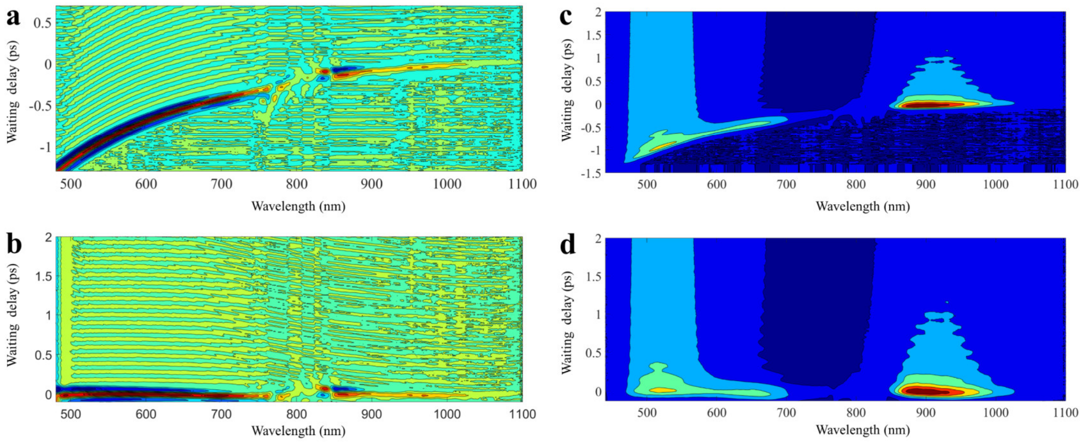

Supplementary Figure 18. (**a, b**) Differential absorbance maps of a CaF_2 substrate with uncorrected and corrected chirp, respectively. (**c, d**) Differential absorbance maps of $Ti_3C_2T_x$ substrate with uncorrected and corrected chirp, respectively.

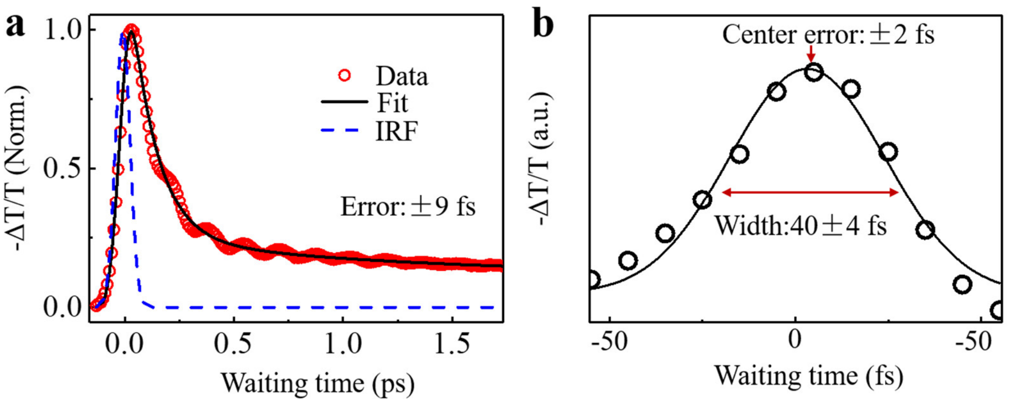

Supplementary Figure 19. Experimental error analysis. (**a**) Measured data (circle) and fitting result (solid line) at pump 780 nm probe 900 nm with deconvoluted in $Ti_3C_2T_x$. (**b**) A measured instrument response function (IRF) signal in a substrate of CaF_2 was fitted with a Gaussian function.

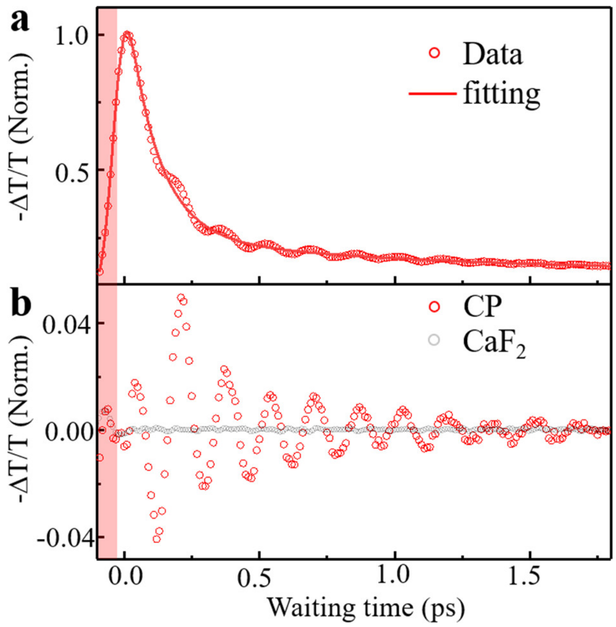

Supplementary Figure 20. (**a**) Data of $Ti_3C_2T_x$ at pump 780 nm probe 900 nm and fitting result with deconvoluted. (**b**) Compared data of CP data and the background signal of CaF_2.

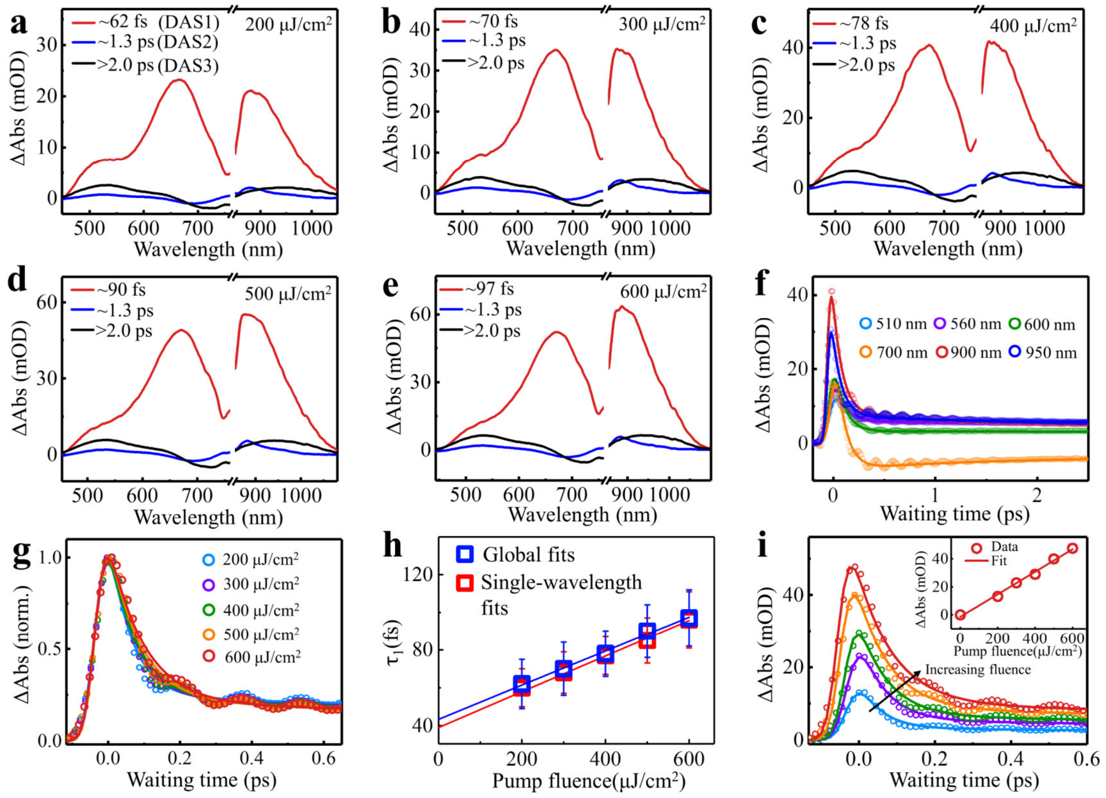

Supplementary Figure 21. The global fits for the $Ti_3C_2T_x$ film under the excitation of 780 nm. (**a-e**) Acquired decay-associated spectra (DAS) from the global fits on the transient data at different pump fluences. The absence of spectra ranging from 760 to 860 nm is attributed to the reason that the fundamental pulse leads to strong noise. (**f**) Selected dynamics trace monitored at indicated wavelengths and corresponding fitting results. (**g**) the normalized pump fluence-dependent dynamics data (circle) probed at 910 nm and the corresponding global fitting results (lines). (**h**) Comparison of the fitted time constants (τ_1) using the global and single-wavelength analysis. The error bars represent standard deviation. The error bars represent standard deviation. (**i**) The pump fluence-dependent of transient signal probed at 910 nm, and the inset exhibits the data of signal size (circles) at peak and a linear fitting result (line).

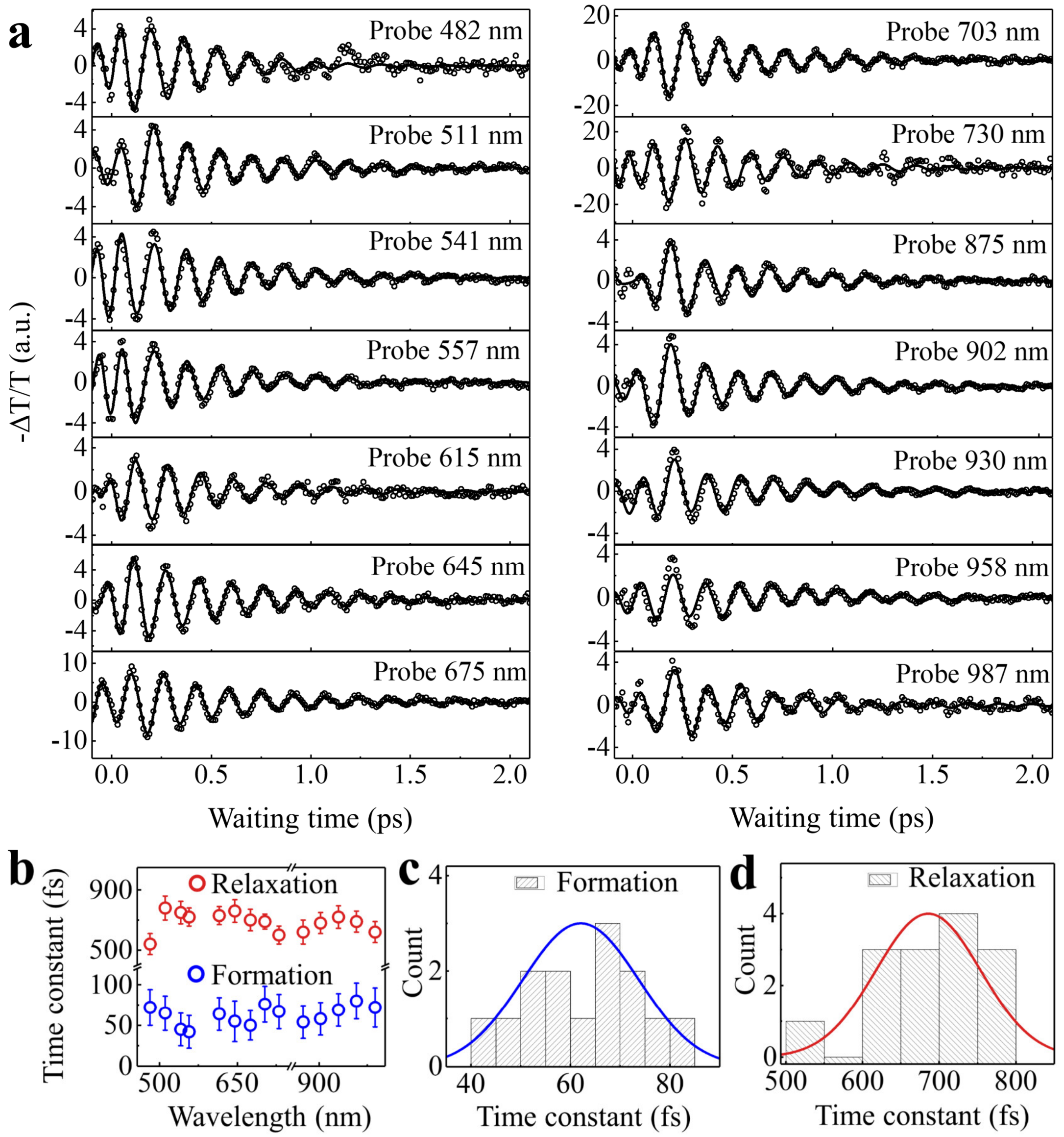

Supplementary Figure 22. Analysis of oscillatory components via fitting multi-probe-wavelengths. (**a**) Oscillatory data (circles) and fitting results (lines) at indicated probe wavelengths. (**b**)The Probe-wavelength dependent formation and relaxation time constants. The error bars represent standard deviation. (**c**) The distribution of formation time constants (**d**) The distribution of relaxation time constants.

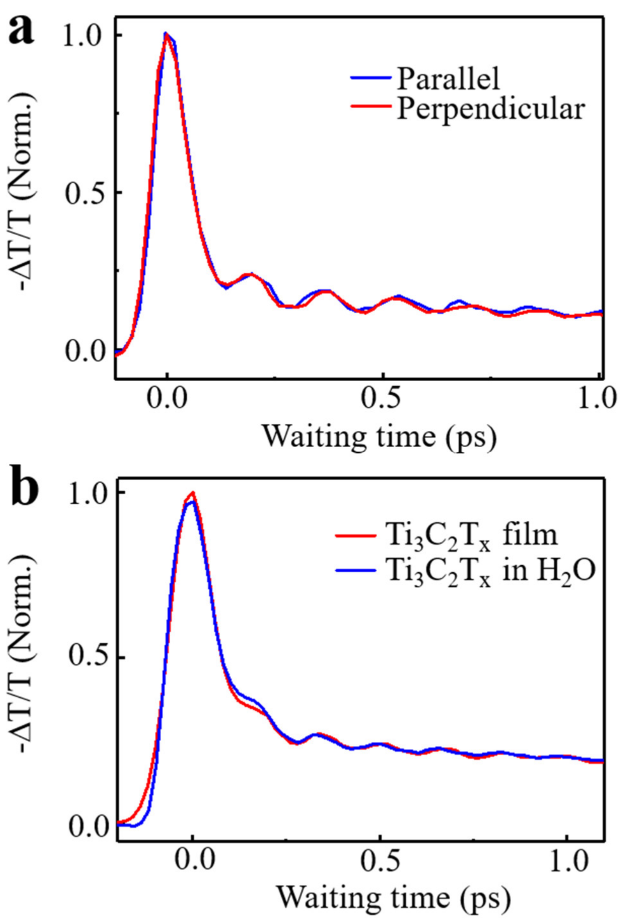

Supplementary Figure 23. (**a**) Pump-probe data of $Ti_3C_2T_x$ film for parallel and perpendicular polarization. (**b**) Pump-probe data of $Ti_3C_2T_x$ film and $Ti_3C_2T_x$ in H_2O at pump 780 nm (500 μJ/cm^2) probe 900 nm.

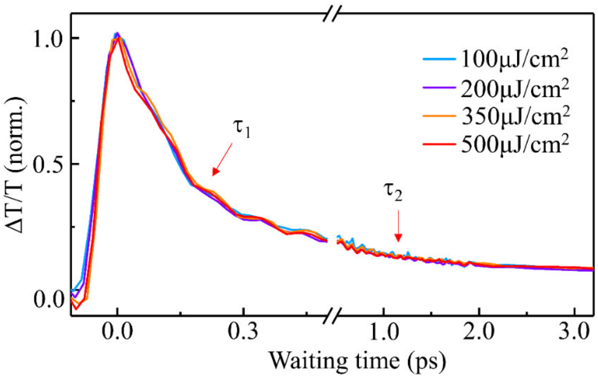

Supplementary Figure 24. Pump 700 nm fluences-dependent dynamics of Mo_2CT_x were monitored at 550 nm.

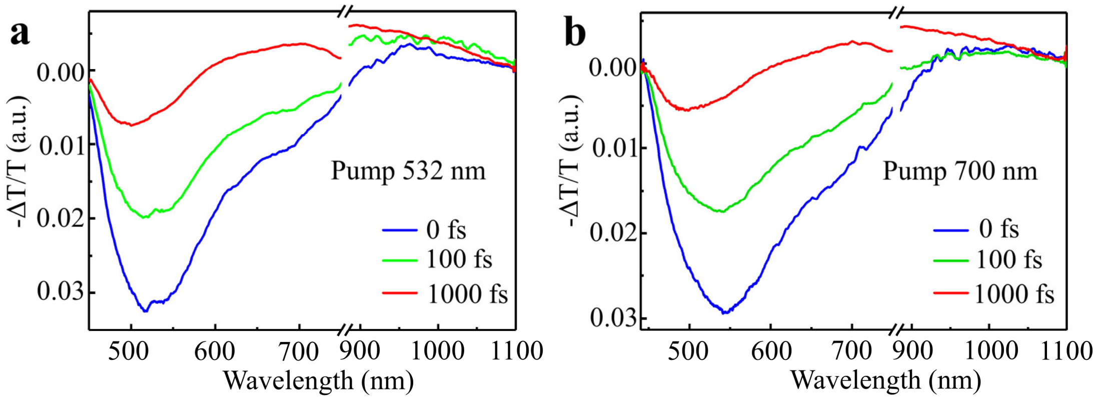

Supplementary Figure 25. Transient spectra of Mo_2CT_x at various waiting times with two pump wavelengths. (**a**) Pump 532 nm. (**b**) pump 700 nm.

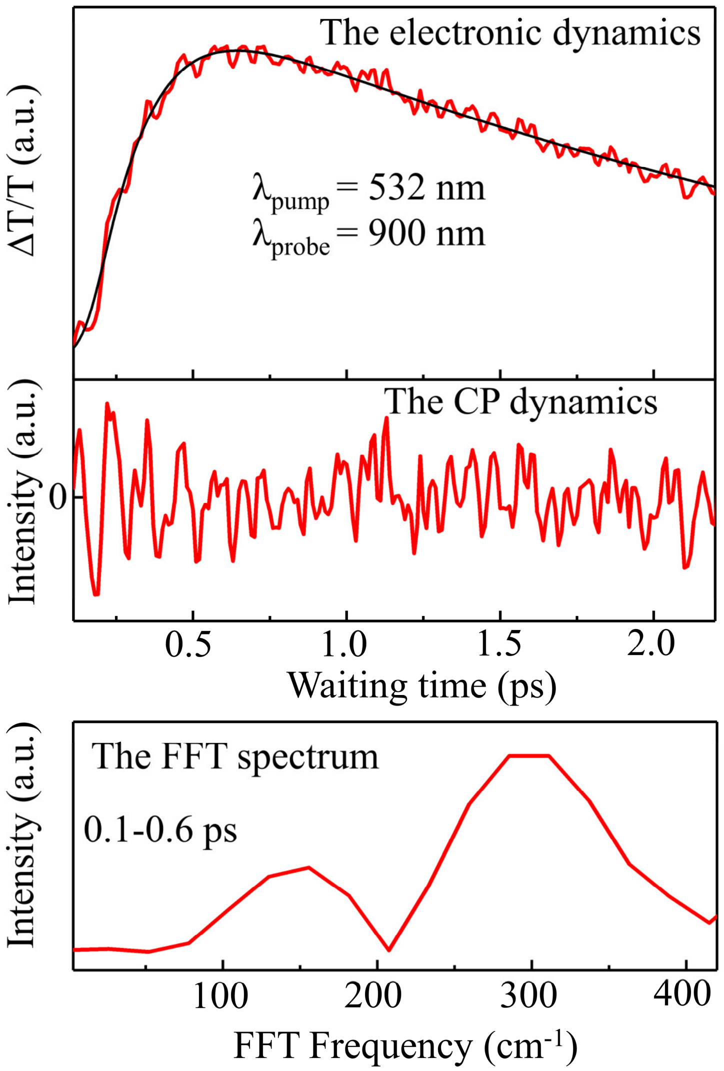

Supplementary Figure 26. The electronic, CP dynamics, and the FFT spectrum of Mo_2CT_x were monitored at 930 nm with pumping at 532 nm.

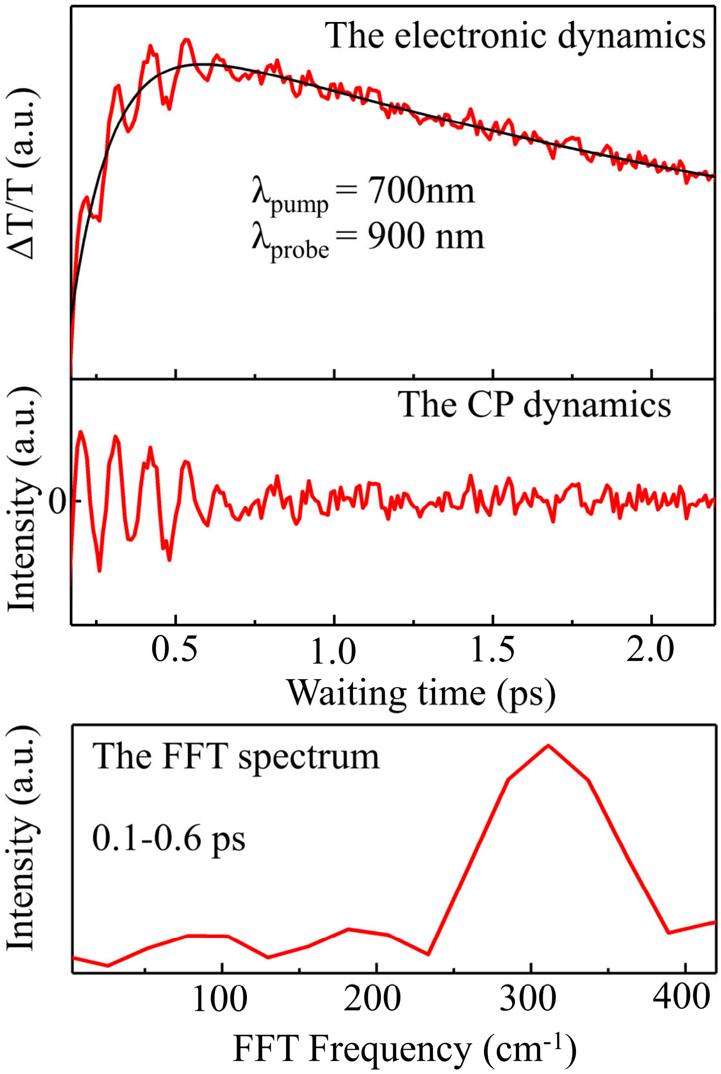

Supplementary Figure 27. The Electronic and CP dynamic and FFT spectrum of Mo_2CT_x were monitored at 930 nm with pumping at 700 nm.

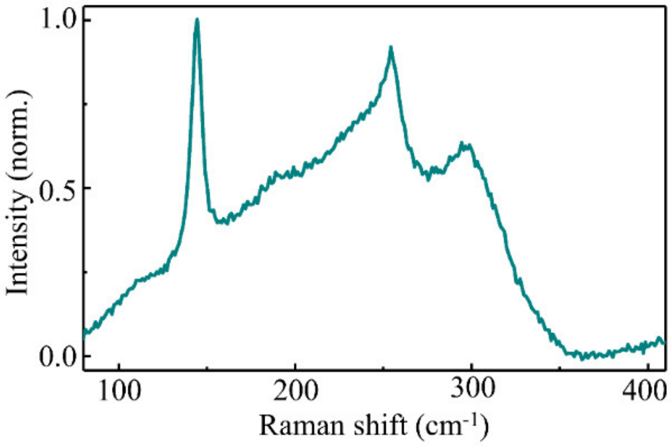

Supplementary Figure 28. The Raman spectrum of Mo_2CT_x film with 532 nm excitation.

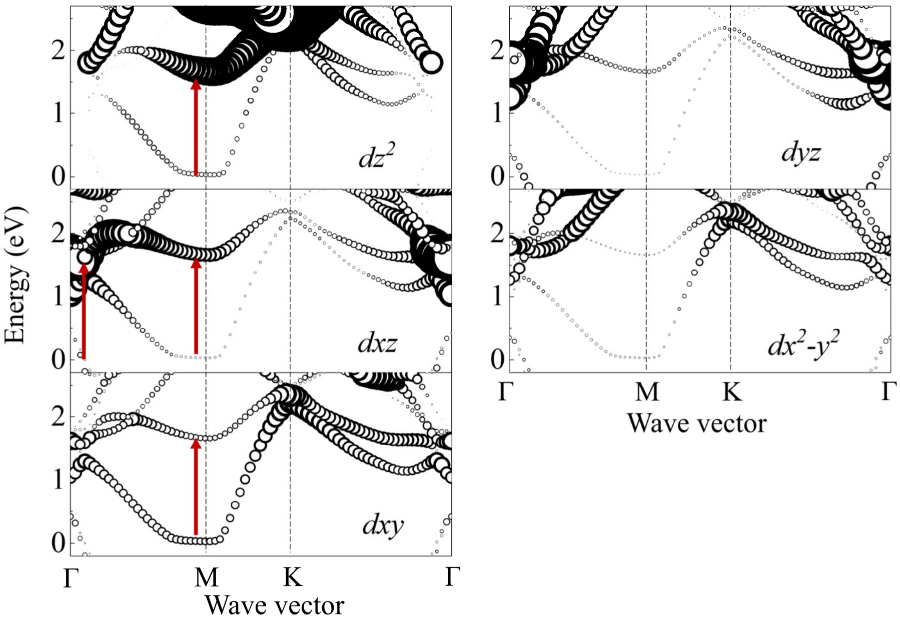

Supplementary Figure 29. Hybrid functional fatband of d-ortials of $Ti_3C_2T_x$ (The diameters of the circles represent the intensity of DOS). The result showed the minimal contribution of *dyz* and dx^2-y^2 orbitals for transient with excitation at SP band. The red arrows indicate possible electronic transitions after excitation.

Supplementary Table 1: List of fluence-dependent electronic relaxation time constants for $Ti_3C_2T_x$ with pumping at 780 nm and 532 nm.

Pump 780 nm	τ_1 (fs)	τ_2 (fs)
200 μJ/cm^2	60±11	1250±100
300 μJ/cm^2	68±10	1290±130
400 μJ/cm^2	77±12	1270±160
500 μJ/cm^2	95±15	1250±140
600 μJ/cm^2	105±15	1270±170

Pump 532 nm	τ_1 (fs)	τ_2 (fs)
200 μJ/cm^2	92±12	1410±280
335 μJ/cm^2	109±15	1260±225
500 μJ/cm^2	118±15	1320±241
630 μJ/cm^2	153±13	1380±210

Supplementary Table 2. List of time constants relating to electronic and phonon dynamics for $Ti_3C_2T_x$ with pumping at 780 and 532 nm.

$Ti_3C_2T_x$	Nonthermal electron decay time constant (fs)	A_{1g} mode formation time constant (fs)	A_{1g} mode relaxation time constant (fs)
Pump 532 nm	57±10	67±20	820±60
Pump 780 nm	39±10	55±20	680±42

Supplementary Table 3. List of fluence-dependent CPs relaxation time constants for $Ti_3C_2T_x$ with pumping at 780 nm and 532 nm.

Pump 780 nm	A_{1g} mode relaxation time constant (fs)	E_g mode relaxation time constant (fs)
200 μJ/cm^2	760±45	1280±470
300 μJ/cm^2	730±43	1230±420
400 μJ/cm^2	700±48	1200±490
500 μJ/cm^2	680±42	1180±440
600 μJ/cm^2	640±49	1140±460

Pump 532	A_{1g} mode relaxation time constant (fs)	E_g mode relaxation time constant (fs)
200 μJ/cm^2	960±50	None
335 μJ/cm^2	890±55	None
500 μJ/cm^2	820±60	None
620 μJ/cm^2	680±70	None

Supplementary Table 4. List of time constants relating to electronic and phonon dynamics for $Ti_3C_2T_x$ with pumping at 380, 1300, and 1580 nm.

$Ti_3C_2T_x$	τ_1 (fs)	τ_2 (fs)	A_{1g} mode relaxation time constant (fs)
Pump 380 nm	115±20 fs	1170±159	580±40 fs
Pump 1300 nm	80±15 fs	1190±200	690±55 fs
Pump 1580 nm	126±15 fs	1320±240	685±50 fs

Supplementary Table 5. List of fluence-dependent electronic relaxation time constants for Mo_2CT_x with pumping at 700 nm.

Mo_2CT_x	τ_1 (fs)	τ_2 (fs)
100 μJ/cm^2	155±25	1270±300
200 μJ/cm^2	158±23	1430±350
350 μJ/cm^2	163±22	1610±400
500 μJ/cm^2	167±24	1720±300

Supplementary Table 6. List of time constants relating to electronic and phonon dynamics for Mo_2CT_x with pumping at 532 and 700 nm.

Mo_2CT_x	Nonthermal electron decay time constant (fs)	CP formation time constant (fs)	CP relaxation time constant (fs)
Pump 532 nm	155±10	<45	890±50
Pump 700 nm	160±15	163±30	870±55

Supplementary Note 1: The transient spectra and probe dependent optical phonon distribution and phase shifts in $Ti_3C_2T_x$.

The transient spectra of $Ti_3C_2T_x$ with pumping 532 nm and 780 nm (Supplementary Fig. 3) exhibit similar spectral features, which are a negative bleaching signal (GBS) in 700-850 nm due to the SP band and two positive excited-state absorptions (ESA) in 450-700 nm and 900-1000 nm wings on both sides of the bleach. The dynamics traces of the GBS at 720 nm and ESA at 510, 650, and 900 nm were selected because these four distinct brands showed smaller overlap between GBS and ESA compared with other wavelengths. The transient spectral detected window were divided into three t regions based on spectral response: (1) the inter-band electron transient response 450-700 nm, (2) 700-850 nm near the SP, (iii) the free-electron response 900-1000 nm[1]. Besides, there is a striking oscillating characteristic superimposed on electronic dynamics. The electronic dynamics response was different for every detected spectral region. Supplementary Fig. 4a & b, and Supplementary Fig. 5a & b showed pump-probe data and fitting. The related electronic dynamics of $Ti_3C_2T_x$ in Supplementary Fig. 6 were fitted by two exponential decays respectively at probe 900 nm. The results showed that most of electronic energy dropped to some CPs due to strong electron-CPs coupling in 100 fs. The remaining energy dissipated lasting ~1.3 ps by thermal electron-acoustic phonons coupling or optical phonon-acoustic phonons coupling. The relative slow part had not been observed in noble metals but had been reported in graphene[2, 3] and graphite[4, 5]. The FFT spectra of both the experimental data are shown in Supplementary Figs. 4d and 5d. The mode (~200 cm^{-1}) was observed with pumping 532 nm and 780

nm at probe 510, 650, 720 nm. In contrast, two modes were observed only at probe 900 nm with pumping 780 nm (Supplementary Fig. 4d). These results indicate that the A_{1g} mode plays a significant role in the electron-phonon coupling process, while the coupling between electrons and E_g phonons is weaker.

One interesting issue is that the coherent oscillations of $Ti_3C_2T_x$, an identical frequency at ~200 cm^{-1} at the ESA region (510 nm and 900 nm, Supplementary Fig. 4c) and near plasmon band bleach region (650-720 nm, Supplementary Fig. 4c & f), carry the opposite phases, which corresponds to a phase shift of ~π rad. In other words, after optical pulse excited $Ti_3C_2T_x$ nanosheets, the coherent signals at ESA peaks are gradually increased, but the coherent signals at the plasmon state are gradually decreased. The time-dependent coherent dynamics show that the coherent signal at the plasmon state decreases while the coherent signal at the ESA state increases. It suggests that the vibrational wave packet of coherent phonons is transferred back and forth between the ESA state and the plasmon state. Similar results were also found in molecular and cluster systems[6, 7].

Supplementary Note 2: The time constants of electron-electron scattering in plasmonic materials.

To provide information on the energy branching into electron-electron scattering and the population of coherent phonons from non-thermalized electrons, we obtained the electron-electron scattering half-time of ~50 fs in $Ti_3C_2T_x$ and ~125 fs in gold nanorods (GNRs, characterizations as shown in Supplementary Figs. 7a) from transient

absorption (TA) spectra as shown in Supplementary Fig. 8a & b. The methods to achieve the above values are as follows:

The pump-probe technique investigates electron-electron scattering using a laser pulse to excite electrons and measure the optical response in plasmonic materials. After absorbing photons, the electron temperature increases *via* electron-electron scattering to change the dielectric function, resulting in the SP band to blueshift and broaden within ~100 fs. Then, the SP band shifts back and becomes narrow on the ps-ns timescale as the electron temperature cools down[8, 9]. The observed time-dependent transient spectra feature of GNRs (Supplementary Fig. 7b) is similar to the previous reports[8, 9]. Hence, the electron-electron scattering time could be obtained by observing the blue-shifted time of the SP band in TA spectra. In our observation (supplementary Fig.8 a & b), the plasmonic bands in TA spectra showed that the blue-shifted times are ~100 and ~250 fs in $Ti_3C_2T_x$ and GNRs, respectively, indicating the electron-electron scattering half-times of ~50 fs in $Ti_3C_2T_x$ and ~125 fs in GNRs. In addition, another method to get a time constant of electron-electron scattering is to fit a rise dynamic curve[10, 11]. The rise dynamics trace at 903 nm were selected because smaller overlap between GBS and ESA in $Ti_3C_2T_x$. Supplementary Fig.8 c & d showed the rise time of 55 ± 12 fs in $Ti_3C_2T_x$ and 110 ± 20 fs in GNRs. The obtained half-times (~125 and ~50 fs) from the blue-shifted features of TA spectra and rise dynamic time (110 ± 20 and 55 ± 12 fs) are consistent in GNRs and $Ti_3C_2T_x$, Hence, the timescales between electron-electron scattering (~50 fs) and electron-phonon coupling (57±10 and 39±10 fs) are close. And the energy branching should be close in the two physical processes.

Supplementary Note 3: The pump fluence-dependent dynamic evolution of GNRs.

To further confirm the channel III in Fig. 1 in $Ti_3C_2T_x$, a control experiment of GNRs was performed. The pump fluence-dependent dynamics in GNRs were monitored as shown in Supplementary Fig.11 a and the inset. The dynamics showed significant phase shift and delayed appearance time (ΔT≈5 ps) of CP signal with increasing the pump fluence. The supplementary Fig.11 b & c showed different relaxation dynamics after excitation with a low and high pump fluence, which could be fitted with a two-exponential function. The fitting results exhibited different decay time constants (2.5±0.2 and 5.1±0.3 ps). These timescales are commensurate with electron-phonon coupling in gold nanostructures[12, 13].

The dynamics of GNRs could be understood with the two-temperature model[14, 15, 16]. The equations are given as follows:

$$C_e(T_e)\frac{dT_e}{dt} = -g(T_e - T_l) \quad (1)$$

$$C_l\frac{dT_l}{dt} = g(T_e - T_l) \quad (2)$$

T_e and T_l are the temperatures of electron and lattice vibration (phonon), C_e and C_l are the electronic and lattice heat capacities. g is the electron-phonon coupling constant. The electron decay-rate (dT_e/dt) equals to the phonon rise-rate (dT_l/dt). The electron heat capacity: $C_e(T_e) = \gamma T_e$, the γ is the electron heat capacity constant, which depends on electron temperature. The measured electron-phonon interaction time constants in our experiment (Supplementary Fig.11 b and c) should accompany with increasing electron temperature after electron-electron scattering and pump-fluence, consistent with these previous observations [12, 13, 17, 18]. Meanwhile, the phonon rise-time constant

value is also higher with increasing pump fluence. Supplementary Fig.11 d showed the two fitted phonon dynamics results with two pump fluences, indicating that the phonon dynamic traces reach their maximum at ~12 and ~17 ps after excitation with low and high pump fluence, respectively. The difference ($\Delta T \approx 5$ ps) between the two times (12 and 17 ps) is consistent with the delayed appearance time ($\Delta T \approx 5$ ps) of the CP signal in Supplementary Fig.11a (inset). Therefore, the dynamic process in the GRNs corresponds to thermal electron-phonon coupling (The Channel I in Fig. 1).

Supplementary Note 4: The transient spectra and symmetry-dependent excited electron-phonon coupling in Mo_2CT_x.

The transient spectra of Mo_2CT_x (Supplementary Fig. 25) exhibited GBS in 450-900 nm and one positive ESA in 900-1000 nm in 100 fs with pumping 532 nm and 700 nm (~300 μJ/cm^2). With time evolution, the transient spectra of Mo_2CT_x changed to GBS in 450-600 nm and ESA in 650-1000 nm at 1000 fs. The region in 450-600 nm can be assigned to SP band[19]. The dynamics trace of the GBS at 550 nm（Supplementary Fig. 15d）and ESA at 930 nm (Supplementary Fig. 26 and 27）were monitored due to the smaller overlaps between GBS and ESA. Furthermore, we extracted the coherent phonon signals (Supplementary Fig.15a and Supplementary Fig.26 and 27) from the Mo_2CT_x electron dynamics at probe 550 and 930 nm with 532 and 700 nm excitation, respectively.

The time-partitioned FFT spectra of Mo_2CT_x showed the multiple vibration modes with pumping 532 nm. These modes could be observed in both two probing frequencies: 550

nm (Supplementary Fig.15b) and 930 nm (Supplementary Fig.26). The 700 nm excitation could create the broad-band vibration (~300 cm^{-1}) in 0.1-0.6 ps (Supplementary Fig. 15b). With time delay, a narrow band vibration mode (~330 cm^{-1}) appeared in 0.6-2.0 ps (Supplementary Fig. 15b). These vibration modes could also be observed in Raman spectra of Mo_2CT_x film under 532 nm excitation (Supplementary Fig. 28). The vibration mode at ~150 cm^{-1} is attributed to the in-plane vibration mode ($2E_g$) of the Mo_2C and the vibration modes at ~300 and ~330 cm^{-1} are attributed to two vibrational modes in Mo_2CT_x[19].

Similar to $Ti_3C_2T_x$, the analysis for Mo_2CT_x also needs to consider DOS and the matching between electron orbitals and vibrational modes. As shown in Supplementary Fig. 16a, under nonresonant excitation at 700 nm, a-b transitions can occur at Γ and K points. The results of Supplementary Fig. 16b show that in the b band, only dz^2 orbital at Γ and K points have a higher energy density of states, so the electrons are mainly excited to the dz^2 orbitals under SP nonresonant excitation. However, a-c band transitions show that the electrons can be exited to *dxy*, *dzx* and *dyz* orbitals at Γ and M points, and dz^2 orbital at K point under the resonant excitation. Based on the DOS results at the c band in Supplementary Fig. 16b, excited to the dz^2 orbital is still the main route.

Supplementary Note 5: The calculation of the electron–phonon coupling constant λ.

In order to compare the electron-phonon coupling strengths with other materials, we determined the electron-phonon coupling factors and constants, $\lambda\langle\omega^2\rangle$ and λ[20] as

follows:

$\lambda<\omega^2> = 605$ meV^2 and $\lambda = 1.62$ (A_{1g});

$\lambda<\omega^2> = 20$ meV^2 and $\lambda = 0.054$ (E_g);

These values were calculated by combing with the two-temperature model[15] and nonequilibrium model[21].

The electron-phonon coupling strengths are calculated as follows:

According to the two-temperature model (TTM)[15, 21]:

$$\lambda\langle\omega^2\rangle = \frac{\pi}{3}\frac{k_B T_e}{\hbar\tau_{e-ph}} \qquad (3)$$

And the nonequilibrium model (NEM)[21, 22]:

$$\lambda\langle\omega^2\rangle = \frac{2\pi}{3}\frac{k_B T_l}{\hbar\tau_{e-ph}} \qquad (4)$$

T_e is electron temperature. K_B is Boltzmann's constant, $\hbar$ is Planck's constant, and τ_{e-p} is the electron-phonon coupling time constant. We take the average value of electron-phonon coupling time constant for two excitation wavelengths (39 fs with excitation at 780 nm and 57 fs with excitation at pump 532 nm, Fig. 3 c & d): τ_{e-ph} as 50±10 fs for A_{1g} and 1500±500 fs for the E_g (The occurred time constant is 1-2 ps for E_g. We take the intermediate value of 1.5 ps to calculate the coupling strength). The values of electron-phonon coupling strengths ($\lambda\langle\omega^2\rangle$) were obtained as follows:

The TTM model: $\lambda\langle\omega^2\rangle = 403$ meV^2 (A_{1g});

The NEM model: $\lambda\langle\omega^2\rangle = 806$ meV^2 (A_{1g});

The TTM model: $\lambda\langle\omega^2\rangle = 13.5$ meV^2 (E_g);

The NEM model: $\lambda\langle\omega^2\rangle = 27$ meV^2 (E_g).

The TTM is based on the assumption that an electron-electron scattering time constant

($\tau_{e\text{-}e}$) is much shorter than an electron-phonon coupling time constant ($\tau_{e\text{-}ph}$) while the NEM model is applicable when $\tau_{e\text{-}e} > \tau_{e\text{-}ph}$. However, the time constants of $\tau_{e\text{-}e}$ and $\tau_{e\text{-}ph}$ are quite close in $Ti_3C_2T_x$. Therefore, we take the average value of $\lambda<\omega^2>$ in the TTM and the NEM model as 605 meV^2 (A_{1g}) and 20 meV^2 (E_g). The $\langle\omega^2\rangle$ value could use the approximation $\langle\omega^2\rangle = \theta^2_D/2$.[23] The θ_D is Debye temperature, which is 317 K in $Ti_3C_2T_x$[24]. The $\theta^2_D/2$ is 373 meV^2. The calculated electron-phonon coupling constants (λ) are 1.62±0.33 (A_{1g}) and 0.054±0.018 (E_g).

Supplementary Note 6: Data analysis of dynamics for electron and coherent vibration.

It has been reported that the time-dependent transit signals are determined by electronic dynamics in plasmonic systems[8, 25]. In our experiments, the electron population of the SP-MXene above the Fermi level was changed by photoexcitation or injection, which would modify the optical absorption coefficient. From the energy point of view, the decay rate from electrons (N_E) to CP was expressed as K_{cp} and the decay rate from electron (N_E) to low-energy phonons was presented as K_{ap}. Thus, the population of electrons after photoexcitation could be approximately expressed by:

$$\frac{dN_E}{dt} = -N_E * K_{cp}$$

$$\frac{dN_E}{dt} = -N_E * K_{ap}$$

Electronic relaxation signals could be fitted by:

$$S_e(t) = \text{IRF} \otimes (A * e^{-\frac{t}{\tau_1}} + B * e^{-\frac{t}{\tau_2}}) \quad (5)$$

Where $S_e(t)$ is the electron signal intensity with time delay. A and B and τ_1 and τ_2 are amplitudes and time constants of the two exponential decay components. Then the high-

energy electron or SP impulsively induces vibration of atoms. The rapid response rates of coherent phonons are accompanied by the decay rates of electrons or SP. Then coherent phonons relaxation would accompany the excitation of other phonons. The oscillating components signals are well fitted by:

$$S_c(t) = \sum_{i=1,2} A_i \cos(\omega_i t + \theta_i) * \left(-e^{-\frac{t}{\tau_{fi}}} + e^{-\frac{t}{\tau_{ri}}}\right) \quad (6)$$

Where $S_c(t)$ is the oscillating components signals with time delay. A_i is the oscillation amplitude, ω_i is the characteristic frequency of phonon. θ_i is the phase shift. τ_{fi} is the formation/response time constant for appearance excited CP and τ_{ri} is the coherent phonon relaxation constant.

Supplementary Note 7: The correcting the chirp of white light and data processing.

A differential absorbance map with chirp recorded on a CaF_2 substrate is presented in Supplementary Fig.18a. Both the pronounced coherent artifact and the coherent oscillations reveal the chirp of the white light probe[26]. The first step in the data analysis is correcting the differential absorbance map for the wavelength-dependent time zero caused by the probe chirp. The validity of this approach is verified by retrieving the time zero from the phase of off-resonantly generated coherent artifact in a CaF_2 substrate. The white light chirp map can be corrected by means of wavelength-dependent interpolation on a common time axis[27]. Supplementary Fig. 18b shows a result of a CaF_2 by correcting for the chirp in the time-zero of their white light with the pump pulse. Supplementary Fig. 18c & d shows the results of the uncorrected and corrected chirp of $Ti_3C_2T_x$, respectively. The error bars result from the sum of fitting

measured data and the experimental system errors. For example, Supplementary Fig. 19a & b show an error (9 fs) from fitting a $Ti_3C_2T_x$ dynamics with deconvoluted and an error (6 fs, sum of a peak and a width error) from fitting instrument response function. Hence the sum of error is 15 fs. Supplementary Fig. 20a shows a data and fitting result of $Ti_3C_2T_x$ after the pump at 780 nm and monitored at 900 nm. Supplementary Fig. 20b illustrates CP data and a background signal of CaF_2. MXene data in the main text have been subtracted from the background signal.

Supplementary Fig. 23a shows pump-probe parallel and perpendicular polarization for $Ti_3C_2T_x$ film. The data indicate that polarization impacts minimally on coherent signals. Supplementary Fig. 23b shows comparative experimental results in $Ti_3C_2T_x$ film and monolayers $Ti_3C_2T_x$ in H_2O, exhibiting similar coherent and dynamic traces.

Supplementary References

1. Lioi DB*, et al.* Simultaneous ultrafast transmission and reflection of nanometer-thick $Ti_3C_2T_x$ MXene films in the visible and near-infrared: implications for energy storage, electromagnetic shielding, and laser systems. *ACS Appl. Nano Mater.* **3**, 9604-9609 (2020).
2. Johannsen JC*, et al.* Direct view of hot carrier dynamics in graphene. *Phys. Rev. Lett.* **111**, 027403 (2013).
3. Tielrooij KJ*, et al.* Photoexcitation cascade and multiple hot-carrier generation in graphene. *Nat. Phys.* **9**, 248-252 (2013).
4. Kampfrath T, Perfetti L, Schapper F, Frischkorn C, Wolf M. Strongly coupled optical phonons in the ultrafast dynamics of the electronic energy and current relaxation in graphite. *Phys. Rev. Lett.* **95**, 187403 (2005).
5. Breusing M, Ropers C, Elsaesser T. Ultrafast carrier dynamics in graphite. *Phys.*

Rev. Lett. **102**, 086809 (2009).

6. Li S*, et al.* Real-time visualization of the vibrational wavepacket dynamics in electronically excited pyrimidine via femtosecond time-resolved photoelectron imaging. *J. Chem. Phys.* **147**, 044309 (2017).
7. Zhou M, Jin R, Sfeir MY, Chen Y, Song Y, Jin R. Electron localization in rod-shaped triicosahedral gold nanocluster. *Proc. Natl. Acad. Sci. U. S. A.* **114**, E4697-E4705 (2017).
8. Brown AM, Sundararaman R, Narang P, Schwartzberg AM, Goddard WA, Atwater HA. Experimental and ab Initio ultrafast carrier dynamics in plasmonic nanoparticles. *Phys. Rev. Lett.* **118**, 087401 (2017).
9. Rotenberg N, Caspers JN, van Driel HM. Tunable ultrafast control of plasmonic coupling to gold films. *Phys. Rev. B* **80**, 245420 (2009).
10. Voisin C*, et al.* Size-dependent electron-electron interactions in metal nanoparticles. *Phys. Rev. Lett.* **85**, 2200-2203 (2000).
11. Del Fatti N, Voisin C, Achermann M, Tzortzakis S, Christofilos D, Vallée F. Nonequilibrium electron dynamics in noble metals. *Phys. Rev. B* **61**, 16956-16966 (2000).
12. Link S, El-Sayed MA. Spectral properties and relaxation dynamics of surface plasmon electronic oscillations in gold and silver nanodots and nanorods. *J. Phys. Chem. B* **103**, 8410-8426 (1999).
13. Zhou M*, et al.* Evolution from the plasmon to exciton state in ligand-protected atomically precise gold nanoparticles. *Nat. Commun.* **7**, 13240 (2016).
14. Schoenlein RW, Lin WZ, Fujimoto JG, Eesley GL. Femtosecond studies of nonequilibrium electronic processes in metals. *Phys. Rev. Lett.* **58**, 1680-1683 (1987).
15. Brorson SD*, et al.* Femtosecond room-temperature measurement of the electron-phonon coupling constant in metallic superconductors. *Phys. Rev. Lett.* **64**, 2172-2175 (1990).
16. Brorson SD, Fujimoto JG, Ippen EP. Femtosecond electronic heat-transport dynamics in thin gold films. *Phys. Rev. Lett.* **59**, 1962-1965 (1987).

17. Tagliabue G*, et al.* Ultrafast hot-hole injection modifies hot-electron dynamics in Au/p-GaN heterostructures. *Nat. Mater.* **19**, 1312-1318 (2020).
18. Park S, Pelton M, Liu M, Guyot-Sionnest P, Scherer NF. Ultrafast resonant dynamics of surface plasmons in gold nanorods. *J. Phys. Chem. C* **111**, 116-123 (2007).
19. Velusamy DB*, et al.* MXenes for plasmonic photodetection. *Adv. Mater.* **31**, 1807658 (2019).
20. Allen PB. Theory of thermal relaxation of electrons in metals. *Phys. Rev. Lett.* **59**, 1460-1463 (1987).
21. Gadermaier C*, et al.* Electron-phonon coupling in high-temperature cuprate superconductors determined from electron relaxation rates. *Phys. Rev. Lett.* **105**, 257001 (2010).
22. Lin Z, Zhigilei LV, Celli V. Electron-phonon coupling and electron heat capacity of metals under conditions of strong electron-phonon nonequilibrium. *Phys. Rev. B* **77**, 075133 (2008).
23. Kabanov VV, Alexandrov AS. Electron relaxation in metals: Theory and exact analytical solutions. *Phys. Rev. B* **78**, 174514 (2008).
24. Khaledialidusti R, Anasori B, Barnoush A. Temperature-dependent mechanical properties of $Ti_{n+1}C_nO_2$ (n = 1, 2) MXene monolayers: a first-principles study. *Phys. Chem. Chem. Phys.* **22**, 3414-3424 (2020).
25. Brown AM, Sundararaman R, Narang P, Goddard WA, Atwater HA. Ab initio phonon coupling and optical response of hot electrons in plasmonic metals. *Phys. Rev. B* **94**, 075120 (2016).
26. Dobryakov AL, Kovalenko SA, Ernsting NP. Electronic and vibrational coherence effects in broadband transient absorption spectroscopy with chirped supercontinuum probing. *J. Chem. Phys.* **119**, 988-1002 (2003).
27. Liebel M, Schnedermann C, Wende T, Kukura P. Principles and applications of broadband impulsive vibrational spectroscopy. *J. Phys. Chem. A* **119**, 9506-9517 (2015).
